# The roles of m6A methylation in cervical cancer: functions, molecular mechanisms, and clinical applications

**DOI:** 10.1038/s41419-023-06265-2

**Published:** 2023-11-11

**Authors:** Zhonghao Mao, Bingyu Wang, Teng Zhang, Baoxia Cui

**Affiliations:** 1 Cheeloo College of Medicine, Shandong University, No. 44 Wenhua West Road, Jinan City, 250012 Shandong Province China; 2 Department of Obstetrics and Gynecology, Qilu Hospital of Shandong University, No. 107 Wenhua West Road, Jinan City, 250012 Shandong Province China

**Keywords:** Cancer epigenetics, Cervical cancer, RNA modification, Cancer microenvironment

## Abstract

Cervical cancer (CC) is a gynecological neoplasm with the highest incidence rate, primarily attributed to the persistent infection of high-risk Human papillomavirus (HPV). Despite extensive research, the pathogenesis of CC remains unclear. N6-methyladenosine (m6A) methylation, the most prevalent form of epigenetic modification in RNA, is intricately linked to cell proliferation, metastasis, metabolism, and therapeutic resistance within the tumor microenvironment (TME) of CC. The involvement of the writer, reader, and eraser in m6A modification impacts the advancement of tumors through the regulation of RNA stability, nuclear export, translation efficiency, and RNA degradation. Here, we discuss the biogenesis of m6A, the atypical expressions of m6A regulators, the mechanisms of molecular interactions, and their functions in CC. Furthermore, we elucidate m6A modification of non-coding RNA. In the context of precision medicine, and with the advancements of genomics, proteomics, and high-throughput sequencing technologies, we summarize the application of m6A in the clinical diagnosis and treatment of CC. Additionally, new perspectives on detection methods, immune regulation, and nano-drug development are presented, which lay the foundation for further research of m6A and provide new ideas for the clinical treatment of CC.

## Facts

- m6A modification mediates multiple biological functions such as sustaining proliferative signaling, deregulating cellular metabolism, and avoiding immune destruction.
- The m6A modification level of RNA in cervical cancer is increased, which regulates tumorigenesis and progression.
- m6A modification occurs extensively in mRNAs and non-coding RNAs, including circRNAs, lncRNAs, and miRNAs.

## Open questions

- What biological processes are involved in the pathogenesis of cervical cancer?
- What role does m6A modification play in the pathogenesis of cervical cancer?
- Does m6A modification have practical application prospects in the diagnosis and treatment of cervical cancer?

## Introduction

Cervical cancer (CC) is the fourth most frequently diagnosed cancer and the fourth leading cause of cancer death in women, with an estimated 606,000 new cases and 342,000 deaths worldwide in 2020 [1]. The persistent infection of high-risk Human papillomavirus (HPV) causes 97% of CC [2]. Treatment strategies for CC patients vary according to FIGO stage and lymph node status [3], and accurate gene diagnosis, targeted therapy, and immunotherapy have become new tumor diagnosis and treatment models. Although the recent inclusion of targeted therapy and immunotherapy is a breakthrough, a significant proportion of patients don’t benefit significantly from vascular endothelial growth factor (VEGF) [4] and immune checkpoint inhibitors (ICIs) [5]. Therefore, patients with persistent, recurrent, and metastatic CC still face significant challenges in clinical treatments and prolonged survival. In conclusion, there is an urgent need to discover and apply some new therapeutic targets to improve the prognosis of patients with CC.

**Fig. 1:**
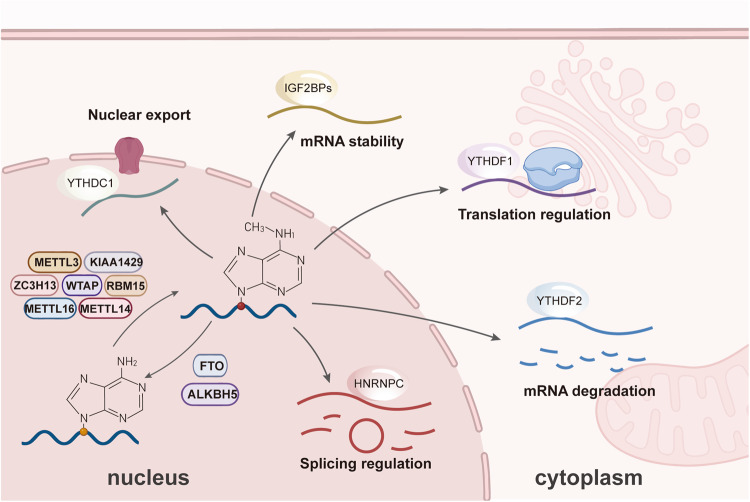
The biogenetic process and mechanisms of m6A methylation modification of RNA in eukaryotes. m6A modification is a dynamic and reversible post-transcriptional process that is biologically regulated by methyltransferases (METTL3, METTL14, METTL16, WTAP, ZC3H13, RBM15, KIAA1429) and demethylases (FTO, ALKBH5). Some RNA-binding proteins (YTHDCs, YTHDFs, IGF2BPs, HNRNPs) bound to the corresponding base at the m6A site play an essential role in regulating RNA stability (IGF2BPs, etc.), nuclear export (YTHDC1, etc.), translation efficiency (YTHDF1, etc.), splicing process (HNRNPC, etc.), and RNA degradation (YTHDF2, etc.).

Epigenetic modification is a chemical modification that occurs on large molecules such as nucleic acids [6] and proteins [7]. Among them, N6-methyladenosine (m6A) is the most common and abundant RNA modification in eukaryotes (accounting for 60% of RNA methylation modification) [8]. Since the advent of the high-throughput sequencing method MeRIP-Seq, genetic and biochemical research in 2012 [9], people’s understandings of m6A modification have had a revolutionary breakthrough. Although the exact location of the m6A site cannot be effectively identified by multiple techniques developed so far, it is generally accepted that it is mainly distributed in the RRACH sequence (R = A or G, H = A, C or U), such as the stop codon and the 3′ untranslated region (3′ UTR) [10]. M6A modification is a dynamic and reversible post-transcriptional process that is biologically regulated by methyltransferases (“writer”) and demethylases (“eraser”) [11]. Some RNA-binding proteins (“reader”) bind to the corresponding base at the m6A site and thus play an essential role in regulating the expressions and functions of RNA. The biogenetic process and mechanisms of m6A methylation modification of RNA in eukaryotes are presented in Fig. 1.

Various studies have shown that the unbalanced expressions of m6A regulators and abnormal m6A modification of critical RNAs play a crucial role in cancer. In this review, we first elucidated the biological process and mechanisms of m6A modification. Furthermore, we summarized abnormal m6A modification events and their effects on the malignant behavior of CC, including mRNA and non-coding RNA (ncRNA). Finally, we discussed the clinical potential of m6A as a biomarker and therapeutic target in CC.

## m6A regulators

**Fig. 2:**
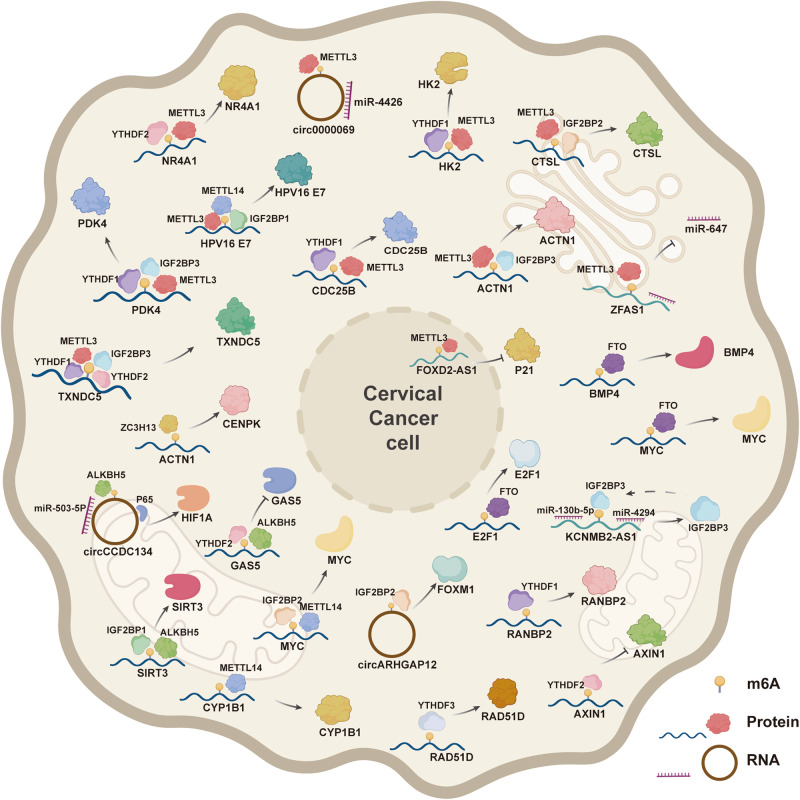
The interaction between m6A regulators and target RNAs in cervical cancer. m6A regulators, including writer, reader, and eraser, bind to the m6A sites of specific target RNAs (including mRNA and ncRNA) and regulate post-transcriptional modification levels and RNA metabolism of specific key genetic material. These m6A-modified transcripts include mRNA (PDK4, CDC25B, HK2, NR4A1, TXNDC5, ACIN1, CTSL, CYP1B1, CENPK, SIRT3, MYC, β-catenin, BMP4, HPV E7, MYC, RANBP2, AXIN1, RAD51D), circRNA (circCCDC134, circARHGAP12, circ0000069), and lncRNA (FOXD2-AS1, ZFAS1, GAS5-AS1, KCNMB2-AS1).

There is growing evidence that abnormal expressions of critical molecules in CC are regulated by extensive m6A modifications, which rely on “writer”, “eraser”, and “reader”. The classical m6A regulators discovered so far are listed in Table 1. Different from the m6A methylation modification of DNA [12], m6A regulators of RNA are diverse and play a role in promoting or suppressing CC in the biological process by targeting different types of RNA and various signaling pathways. So far, studies have found that there were quite a number of abnormal over-expressions of m6A regulatory factors in CC, including methyltransferase-like 13 (METTL3) [13], methyltransferase-like 14 (METTL14) [14], zinc finger CCHC type containing 13 (ZC3H13) [15], insulin-like growth factor 2 mRNA binding proteins (IGF2BP1/2/3) [16–18], YTH domain families (YTHDF1/2/3) [19–21], and fat and obesity-related protein (FTO) [22]. In addition, abnormal downregulation of AlkB homolog 5 (ALKBH5) [23] also affected the progression of CC. The interaction between m6A regulators and target RNAs in CC is shown in Fig. 2.

**Table 1:** The classical functional roles of m6A regulators in RNA metabolism.

Type	m6A Regulator	Function	Reference
writer	METTL3	Catalyzes m6A modification	[25]
	METTL14	Assists METTL3 to recognize the subtract	[26]
	WTAP	Promotes METTL3-METTL14 heterodimer to the nuclear speckle	[27]
	ZC3H13	Bridges WTAP to the mRNA-binding factor Nito	[28]
	RBM15	Binds the m6A complex and recruit it to special RNA site	[29]
reader	IGF2BP1/2/3	Enhances mRNA stability	[40]
	YTHDC1	Promotes RNA splicing and translocation	[41]
	YTHDC2	Enhances the translation of target RNA	[42]
	YTHDF1	Promotes mRNA translation	[43]
	YTHDF2	Reduces mRNA stability	[44]
	YTHDF3	Mediates the translation or degradation	[44]
	HNRNPC	Mediates mRNA splicing	[43]
	HNRNPA2B1	Promotes primary microRNA processing	[45]
eraser	FTO	Removes m6A modification	[52]
	ALKBH5	Removes m6A modification	[53]

### writer

The primary function of m6A “writer” is to initiate the methylation modification of RNA, including METTL3, METTL14, and Wilms tumor 1-associated protein (WTAP), which are thought to control the installation of m6A methylation by forming methyltransferase complex (MTC) [24]. METTL3, initially identified in eukaryotes, is the most critical catalytic element in MTC [25]. METTL14 helps METTL3 identify substrates by taking advantage of its structural advantages [26]. WTAP is responsible for binding to the METTL3-METTL14 heterodimer and stabilizing its structure, thus ensuring its accurate location in the nuclear spot and triggering its catalytic activity [27]. In addition to the above classic m6A writer, other members of MTC have been consecutively found by researchers, such as ZC3H13, RNA-binding motif protein 15 (RBM15) with its paralogue RBM15B. The primary function of ZC3H13 is to maintain the structure and activity of the methyltransferase complex located in the nucleus [28]. In contrast, RBM15/15B acts synergically with the METTL3-WTAP complex to promote the recruitment of methyltransferase complex to the U-rich region of RNA [29].

The modification level of RNA m6A in CC is significantly higher than that in normal cervical tissues [30, 31], which is closely related to the abnormal expressions of the writer in tumor cells. CDC25B, an overexpressed oncogene in tumors, was considered as a “trigger” phosphatase and associated with the G2/M transition [32]. A recent study implied that NR4A1 played an anti-tumor role due to its inducement of cell apoptosis in CC [33]. METTL3 promoted the expression of CDC25B by binding to the m6A site of its mRNA [34] and inhibited the expression of NR4A1 by increasing the m6A level of NR4A1 mRNA, activating the ATK signaling pathway [35]. Additionally, HPV, the major carcinogen of CC, mainly depends on the sustained expressions of virus early protein 6/7 (E6/7) [36]. METTL14 bound to E7 mRNA in an m6A-dependent manner and promoted E7 expression [16]. Immunohistochemistry has revealed that CYP1B1 was expressed in the majority of the CC samples (91/100, 91.0%) but not in normal healthy cervical samples [37]. And it was found that METTL14 enhanced the m6A level of CYP1B1 mRNA and promoted its expression [14]. Previous studies have manifested links between CENPK and tumor malignancy, such as hepatocellular carcinoma [38]. Recent studies have also shown that ZC3H13 regulated the expression of CENPK by altering the 3 ‘-UTR m6A modification level of CENPK mRNA [15].

### reader

M6A “reader” is a class of RNA-binding proteins that can recognize and specifically bind m6A methylation sites, including IGF2BPs, YTHDCs, YTHDFs, and heteronuclear ribonucleoproteins (HNRNPs; HNRNPA2B1, HNRNPC, and HNRNPG) [39]. Different species, diseases, and RNAs select different m6A “readers” to perform specific biological functions, such as maintaining RNA stability, controlling RNA degradation, and facilitating RNA transport, splicing, and translation processes. The roles of the m6A “reader” in CC are shown in Fig. 1. It is generally believed that IGF2BPs, depending on their K homology (KH) domains, can recognize and interact with m6A modification sites to improve the stability of target RNA or enhance its translation efficiency [40]. The functions of YTHDCs vary according to different subtypes. For example, the function of YTHDC1 is mainly to promote nuclear output and alternative splicing of m6A-modified RNA [41], while YTHDC2 is mainly related to the extension of RNA translation [42]. Current studies and opinions suggest that YTHDFs play a complex and diverse role in the regulation of m6A, which plays an essential role in various aspects [43, 44]. HNRNPs, especially HNRNPA2B1, as a common splicing factor, are mainly responsible for regulating alternative splicing in participating in m6A [43, 45].

IGF2BPs and YTHDFs were the primary overexpressed readers found in CC. Whether YTHDCs and HNRNPs have research value still needs to be further confirmed. HPV E6/E7 is closely related to CC cell proliferation, metastasis, and metabolism. Studies have shown that HPV16/18 E6/E7 could promote the expression of IGF2BP1 and IGF2BP2, and their appearance could in turn bind to the m6A site of E7 [43] and other mRNAs such as PDK4 [13], TXNDC5 [31], CTSL [46], SIRT3 [47], MYC [48]. The same mRNA can also be recognized by multiple readers and play a synergistic role through different mechanisms. PDK4 is the most widely distributed PDK isoform which plays oncogenic roles via Warburg effect in human cancer [49]. IGF2BP3 and YTHDF1 could simultaneously recognize the m6A site of PDK4 mRNA and jointly promote the expression of PDK4 protein by maintaining the stability of PDK4 and promoting the translation efficiency of PDK4, respectively, to play their role in metabolic reprogramming [13]. The dysregulation of TXNDC5 has a bearing on endoplasmic reticulum (ER) oxidative stress [50]. YTHDF1/2 and IGF2BP1/2/3 could simultaneously bind TXNDC5 mRNA, which changed the stemness of CC cells by promoting its expression [31]. Previous studies have verified that some “readers” could enhance the degradation of m6A-modified transcripts [51]. In CC, YTHDF2 was involved in the degradation of NR4A1 transcripts via METTL3-induced m6A, accelerating CESE tumorigenesis [35].

### eraser

The m6A “eraser” is a demethylase that deletes methyl groups and removes the m6A modification to ensure that the m6A modification is dynamic and reversible. The two most widely used demethylases are FTO [52] and ALKBH5 [53]. Although both demethylases remove methylation by oxidizing the m6A site, their specific mechanisms of action are not the same. As the first demethylases to be discovered, FTO converts m6A to N6-hydroxymethyladenosine (hm6A), N6-formyladenosine (f6A), and adenosine in a continuous multi-step process with the participation of iron (II) and α-ketoglutarate (α-KG). In contrast, ALKBH5 can directly remove m6A methylation through a one-step catalytic process mediated by itself. Then, the subcellular localization of their roles is different. FTO can bind to the m6A site of RNA in the cytoplasm and nucleus, whereas ALKBH5 can usually only remove m6A methylation in the nucleus. In addition, a discrepancy has been observed in substrate recognition between FTO and ALKBH5. FTO can remove a variety of RNA methylation modifications, such as m6A, m1A, and m6Am, while ALKBH5, as the specific demethylase of m6A, can only affect the m6A site.

It has been reported that the expression of FTO was upregulated in tissue and cell samples of cervical squamous cell carcinoma (CSCC) and suggested a poor prognosis [22]. As a marker protein of epithelial-mesenchymal transformation (EMT), β-catenin was bound by FTO in an m6A-mediated manner. And FTO positively regulated its mRNA and protein expression by reducing its methylation level [22]. Additionally, MYC, as one of the most widely oncogenes, is involved in the carcinogenic mechanism of HPV [54]. And it could be combined with FTO to promote its expression [55]. In CSCC, SIRT3 modulates acetylation or deacetylation of multifarious key enzymes in mitochondria such as ACC1, thereby maintaining metabolic balance [56]. ALKBH5, with the participation of IGF2BP1, down-regulated the expression of SIRT3 via facilitating SIRT3 mRNA degradation in the way of m6A [47].

## Functions and mechanisms of m6A in CC

**Fig. 3:**
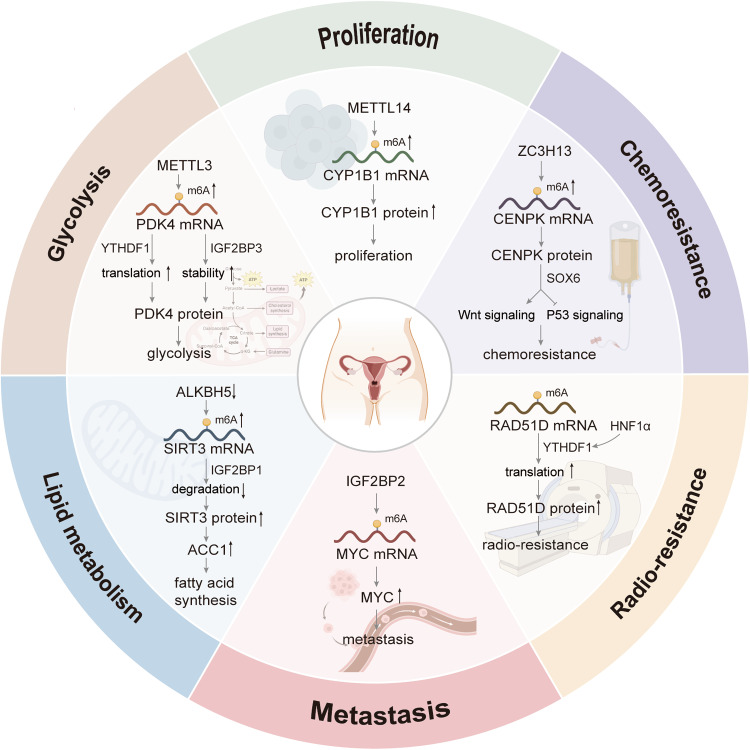
The roles and mechanisms of m6A regulators in cervical cancer. Professor Douglas Hanahan has summarized the typical hallmarks of almost all malignant tumors. As the most abundant epigenetic modification in RNA, m6A plays a vital role in regulating the behaviors of tumor cells including: proliferation (METTL14-CYP1B1 pathway, etc.), metastasis (IGF2BP2-MYC pathway, etc.), glycolysis (METTL3-IGF2BP3/YTHDF1-PDK4 pathway, etc.), lipid metabolism (ALKBH5-IGF2BP1-SIRT3-ACC1 pathway, etc.), chemoresistance (ZC3H13-CENPK-Wnt/P53 signaling pathway, etc.), radio-resistance (YTHDF1-RAD51D pathway, etc.).

The hallmarks of cancer are a set of functional abilities that human cells acquire as they move from normal to a state of tumor growth. Classical cancer hallmarks include sustaining proliferative signaling, activating invasion and metastasis, deregulating cellular metabolism, inducing or accessing vasculature, resisting cell death, and avoiding immune destruction [57, 58]. In 2022, Professor Douglas Hanahan summarized three emerging hallmarks and enabling characteristics, of which non-mutational epigenetic reprogramming is a typical one [59]. Over the years, research and technological advances have revealed important potential mechanisms of epigenetic regulation, represented by RNA m6A methylation modification, in promoting the tumorigenesis of CC [60]. The functions and mechanisms of m6A regulators and m6A-related RNAs in CC are shown in Table 2 and Fig. 3.

**Table 2:** The functions and mechanisms of m6A regulators and m6A-related RNAs in cervical cancer.

Type	Regulator	Role	RNA type	Targets	Mechanism	Function	Ref.
writer	METTL3 ↑	Oncogenic	mRNA	PDK4	Translation regulation, mRNA stability	Glycolysis	[13]
			mRNA	HK2	mRNA stability	Proliferation; Glycolysis	[30]
			mRNA	TXNDC5	mRNA degradation; mRNA stability	Proliferation; Metastasis	[31]
			mRNA	CDC25B	Translation regulation	Proliferation	[34]
			mRNA	NR4A1	mRNA degradation	Proliferation; Migration; Invasion	[35]
			mRNA	CTSL	mRNA stability	Migration; Invasion	[46]
			mRNA	ACIN1	mRNA stability	Proliferation; Migration	[69]
			circRNA	circ0000069	RNA stability	Proliferation; Migration	[97]
			LncRNA	FOXD2-AS1	RNA stability	Proliferation; Migration	[100]
			LncRNA	ZFAS1	Sponging miR-647	Proliferation; Migration; Invasion	[101]
	METTL14 ↑	Oncogenic	mRNA	CYP1B1	Not available	Proliferation; Migration; Invasion	[14]
			mRNA	HPV E7	mRNA stability	Proliferation; Migration	[16]
			mRNA	MYC	Not available	Glycolysis; Proliferation; Migration; Invasion	[48]
	ZC3H13 ↑	Oncogenic	mRNA	CENPK	Not available	Stemness; Chemoresistance; Metastasis; Proliferation	[15]
eraser	ALKBH5 ↓	Antitumor	mRNA	PDK4	Translation regulation, mRNA stability	Glycolysis	[13]
			circRNA	circCCDC134	RNA stability	Proliferation; Migration; Invasion	[23]
			mRNA	SIRT3	mRNA degradation	Migration; invasion; EMT; Lipid metabolism	[47]
			LncRNA	GAS5-AS1	mRNA stability	Proliferation; Migration; Invasion	[70]
	FTO ↑	Oncogenic	mRNA	β-catenin	Not available	Chemoradiotherapy resistance	[22]
			mRNA	MYC	Translation regulation	Proliferation; Migration	[48]
			mRNA	BMP4	Not available	Proliferation; Migration; Invasion	[72]
reader	IGF2BP1 ↑	Oncogenic	mRNA	HPV E7	mRNA stability	Proliferation; Migration	[16]
	IGF2BP2 ↑	Oncogenic	circRNA	circARHGAP12	RNA stability	Proliferation; Migration	[17]
			mRNA	MYC	Not available	Glycolysis; Proliferation; Migration; Invasion	[48]
	IGF2BP3 ↑	Oncogenic	LncRNA	KCNMB2-AS1	RNA stability	Proliferation	[18]
	YTHDF1 ↑	Oncogenic	mRNA	RANBP2	Translation regulation	Proliferation; Migration; Invasion	[19]
	YTHDF2 ↑	Oncogenic	mRNA	AXIN1	mRNA stability	Migration; Invasion; EMT; Chemosensitivity	[20]
	YTHDF3 ↑	Oncogenic	mRNA	RAD51D	Translation regulation	Radio-resistance	[21]

### m6A in cancer cell proliferation, metastasis, and apoptosis

Statistically, approximately 30–40% of patients with CC will relapse during treatment [61]. The reasons lie in the enhanced ability of tumor proliferation, invasion, and metastasis to a certain extent. Mechanistically, these tumor behaviors are regulated by multiple processes including uncontrolled mitosis [62], EMT [63], extracellular matrix (ECM) degradation [64], angiogenesis [65], distal cell-cell communication mediated by exosomes [66], apoptosis [67], and others. However, as a common epigenetic regulatory factor, m6A affects cancer’s malignant behavioral processes.

ACIN1, as a regulator of RNA processing, is abundantly expressed and its high methylation level predicts a high risk of tumors [68]. METTL3 accelerated CC cell growth and migration in cervical carcinoma by reinforcing ACIN1 mRNA stability via an m6A- IGF2BP3-dependent mechanism [69]. As a tumor suppressor in CC, the m6A site of GAS5 mRNA was recognized and silenced by YTHDF2, which promoted the proliferation, migration, and invasion of tumor cells [70]. It was found that BMP4, a fascinating regulator of cancer cell behavior, could promote metastasis in tumors [71]. FTO bound to the N-terminal of BMP4 to form a dimer at the C-terminal in an m6A-dependent manner and regulated the expression, thus promoting the cell proliferation, colony formation, migration, and invasion of CC cells in vitro [72]. Besides, RANBP2 was involved in activating the Wnt/β-catenin pathway in CC [73]. A study has shown that RANBP2 was identified as a critical target of YTHDF1, and YTHDF1 inhibited the apoptosis of tumor cells by regulating the expression of RANBP2 in CC [19].

### m6A in tumor metabolism

In the process of tumor metastasis, metabolic pathways, substrates and products in the tumor microenvironment (TME) constantly change according to the needs of tumor cells, called metabolic reprogramming [74]. There is increasing evidence that dynamic changes in the metabolism of metastatic cells help them adapt bioenergetics during the invasion-metastasis cascade to traverse the mechanically and structurally heterogeneous ECM to escape the primary tumor [75]. Otto Warburg reported on a biological phenomenon known as the Warburg effect [76], in which cancer cells metabolize more glucose and produces more lactate relative to healthy tissues. Through aerobic glycolysis, fuel is provided for the production of ATP, and intermediates and lactate can also be produced to support the biosynthesis of macromolecules, such as amino acids, lipids, and nucleic acids [77]. Similarly, lipid metabolism, such as lipid uptake, synthesis, and hydrolysis, is critical for maintaining cellular homeostasis [78]. Lipids can be used for energy storage and metabolism to maintain rapid cell proliferation and increase reactive oxygen species used by cancer cells to maintain tumor-promoting signaling pathways while avoiding cell death [79].

The enhancement of glycolysis depends on the activity of key enzymes and glucose transporters in aerobic glycolysis to support the increased glucose demand of various cells in TME. In CC, HK2 is a key metabolic enzyme that catalyzes the first reaction of glycolytic pathways by phosphorylating glucose to glucose 6-phosphate [80]. And its expression was significantly upregulated, partly caused by METTL3-YTHDF1 dependent mode, thus enhancing the glycolysis process [30]. And the increased METTL3 expression and the decreased ALKBH5 expression synergically promoted the m6A methylation of PDK4 mRNA. It promoted the translation and stability of PDK4 mRNA in YTHDF1 and IGF2BP3-dependent ways, respectively, and finally increased the expression of PDK4 and promoted glycolysis [13]. In addition to the direct mode of action on crucial glycolysis enzymes, based on METTL14 overexpression, HPV 16/18 E6/E7-dependent IGF2BP2 promoted the expression of MYC mRNA by recognizing the m6A site. It enhanced the effect of MYC on glucose metabolism-related enzymes such as GLUT1, HK2, PFKM, PDK1, and LDHA, thereby indirectly regulating glucose metabolism [48]. ACC1 is a key enzyme that converts acetyl coenzyme A to malonyl coenzyme A. As the upstream of ACC1 in CC, SIRT3 was regulated by m6A of ALKBH5 and IGF2BP1, which could regulate lipid synthesis by promoting the expression of ACC1 [47].

### m6A in therapeutic resistance

For the past few years, the successful transformation from traditional surgery to chemoradiotherapy, targeted therapy, and immunotherapy has represented the thriving position of precision medicine in clinical practice. Unfortunately, resistance to new forms of treatment is now the main reason malignant tumors pose a severe threat to human health and life [81]. For recurrent and metastatic advanced CC, concurrent chemoradiotherapy (CCRT) is still the most important first-line treatment. However, the survival and prognosis of patients are determined by their different degrees of resistance to CCRT. Mechanically, genomic changes are the most common drivers of therapeutic resistance, including altered drug metabolism [82], activation of alternative signaling pathways [83], impaired apoptosis [84], and epithelial interstitial transformation [85].

It has been shown that ZC3H13 [15] and YTHDF2 [20] promoted chemotherapy resistance through m6A and directly affected the prognosis of patients with CC. RAD51D is an essential factor for homologous recombination (HR)-mediated DNA repair [86]. Moreover, YTHDF3 was associated with radiotherapeutic response in CC [21] and further mechanism studies showed that YTHFD3 could bind to the m6A site of RAD51D mRNA, promoting RAD51D translation and participating in homologous recombination and DNA repair.

## Non-coding RNA in m6A

**Fig. 4:**
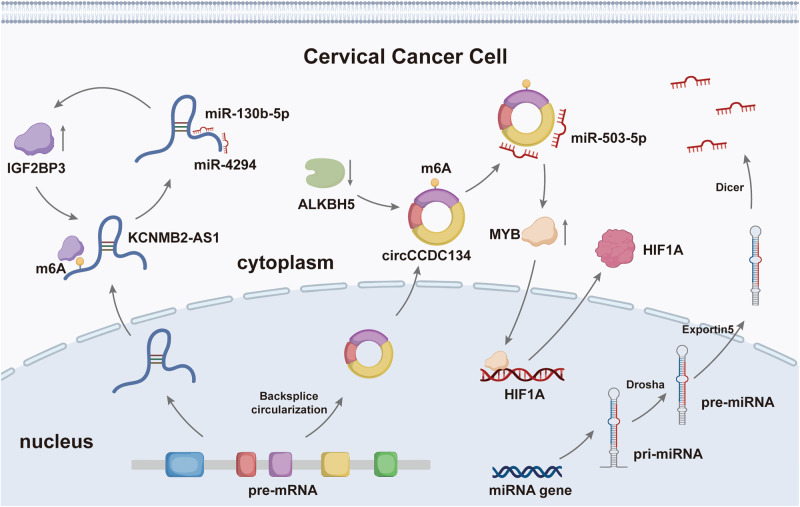
The regulatory role of m6A in non-coding RNA in cervical cancer cells. The most common ncRNA mainly include circRNA, miRNA, and lncRNA, which are widely involved in the progression of cervical cancer. The biogenesis of circRNA relies on a typical back-splicing mechanism, in which the downstream splicing donor connects to the upstream splicing acceptor via one or more exons, generating a covalently closed circRNA. LncRNA is a long-stranded ncRNA which is a product of polymerase II (pol II). miRNA completes its typical biosynthesis pathway through the pri-miRNA/pre-miRNA/miRNA axis under the catalyzation of Drosha, exportin-5, and Dicer. m6A regulators can recognize and function the m6A sites of circRNA and lncRNA, regulating complex ceRNA networks. For example, IGF2BP3 promoted the sponge of miR-130b-5p and miR-4294 by recognizing KCNMB2-AS1, thus improving the expression level of IGF2BP3 and forming a loop. Additionally, circCCDC134 promoted the expression of HIF1A by sponging miR-503-5p and recruiting P65 into the nucleus, respectively.

As the name implies, ncRNA is a class of RNA sequences that do not encode proteins. It has been considered a by-product of genes for quite some time, known as “junk RNA”. However, less than 2% of the genome encodes proteins [87], even though proteins represent genetic information’s end-product and basic function. This has inspired a fever of research by scientists into ncRNA. The most common ncRNA mainly include circular RNAs (circRNAs), micro RNAs (miRNAs), and long non-coding RNAs (lncRNAs), which are widely involved in various human diseases by forming a complex RNA network [88]. Interestingly, with the in-depth study of epigenetics, we found that m6A methylation not only existed on mRNA but also widely occurred on ncRNA [89–91], which was of great significance in regulating the expressions and functions of this unique type of RNA. The regulatory role of m6A in ncRNA in CC cells is shown in Fig. 4.

### circRNA

circRNA is a class of ncRNA molecules with a covalent closed-loop structure [92], and its biogenesis relies on a typical spliceosomal mechanism [93]. For many genes, competition between splicing modes leads to the imbalance of circRNA expressions, which is also a significant cause of tumor occurrence. More importantly, circRNA, once formed, is exceptionally stable and can play a role by accumulating in the cytoplasm through exiting the nucleus [94]. In addition to being localized in the cytoplasm and nucleus, circRNA can also be found in extracellular vesicles in the body’s internal environment [95]. Although the most common mechanism of circRNA is miRNA sponges, protein recruitment, scaffold, and sponges are also fundamental modes of action in which m6a regulators play an important role. Unexpectedly, recent studies have found that some circRNA, although as ncRNA, could encode specific peptides like mRNA and be regulated by m6A [96].

It is found that m6A has multiple regulatory effects on circRNA, including the formation, stable expression, translation, and degradation. In CC, m6A modification could promote the progression of CC by improving the stability of circARHGAP12, circCCDC134, and circ0000069. circARHGAP12 interacted with IGF2BP2 through its m6A site and enhanced FOXM1 mRNA stability, promoting CC cell proliferation and migration by forming circARHGAP12/IGF2BP2/FOXM1 complex [17]. While circCCDC134 promoted the expression of HIF1A by sponging miR-503-5p and recruiting P65 into the nucleus, respectively. Moreover, HIF1A, as a critical regulatory factor in cancer, promoted the proliferation and metastasis of CC cells [23]. In addition, Chen et al. found that in the presence of METTL3, circ0000069 regulated the progression of CC by acting as a molecular sponge of miR-4426 [97].

### lncRNA

LncRNA is a long-stranded ncRNA with more than 200 nucleotides in length, which is a product of polymerase II (pol II) and is highly expressed throughout the cell cycle [98]. LncRNA accounts for about 80% of all ncRNA. According to their different functions, lncRNA can be divided into four types: signal, bait, guide, or scaffold molecules widely involved in vital physiological processes such as metabolism and immunity [99], thus affecting the occurrence or progression of tumors.

In recent years, m6A modification of lncRNA in CC has been found to regulate RNA stability, localization, splicing, and competing endogenous RNA (ceRNA) activity functionally. METTL3 was combined with FOXD2-AS1 to enhance its stability and recruit LSD1 to the promoters of P21 to reduce its expression. As a tumor suppressor gene, P21 was involved in regulating the malignant behavior of CC by the METTL3/FOXD2-AS1/LSD1/p21 axis [100]. In addition, although METTL3 did not promote the expression of ZFAS1, it promoted miR-647 binding to it by enhancing the methylation modification level of ZFAS1, thus promoting the proliferation, migration, and invasion of CC [101]. Zhang et al. also found that IGF2BP3 promoted the sponge of miR-130b-5p and miR-4294 by recognizing KCNMB2-AS1, thus improving the expression level of IGF2BP3 and forming a loop, thus achieving the regulation of CC cell proliferation [18].

### miRNA

miRNA is a non-coding single-stranded RNA molecule encoded by endogenous genes with an average of 22 nucleotides in length [102]. By binding to the 3′ UTR of the target mRNA, it explicitly inhibits the translation of target mRNA molecules, leading to the instability and degradation of the targeted mRNA [103], and thus participates in the regulation of post-transcriptional gene expressions. The typical biosynthetic pathway for miRNA begins with pri-miRNAs, regulating the cell cycle, cell proliferation, apoptosis, angiogenesis, EMT, and tumor invasion.

In 2015, two studies from Rockefeller University on the methyltransferases METTL3 [104] and recognition protein HNRNPA2B1 [105] reported that m6A methylation was involved in miRNA synthesis by modifying pri-miRNA for recognition and processing by DGCR8. Subsequently, more studies have found that multiple m6A methyltransferases, such as METTL14 [106], could similarly affect miRNA synthesis. Large amounts of miRNA were abnormally expressed in CC, including miR-139-3p [107], miR-532-5p [108], and others. Unfortunately, up to now, no studies on m6A-related miRNA have been found in CC. Whether these miRNAs with abnormal expressions and significant effects on tumorigenesis are regulated by m6A is worthy of further investigation.

## Clinical implications of m6A in cervical cancer

**Fig. 5:**
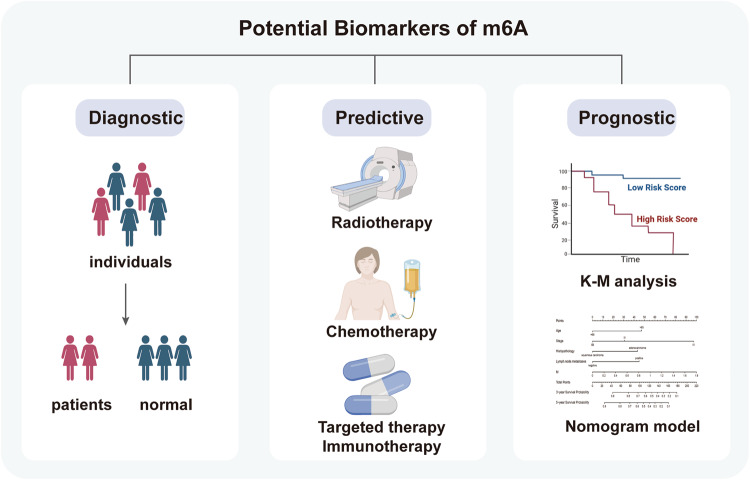
The potential applications of m6A modification as biomarkers in cervical cancer. In cervical cancer, m6A is being revolutionized as potential biomarkers in clinical practice. With the advent of genomics, proteomics, and high-throughput sequencing technologies, m6A has shown good predictive efficacy in terms of diagnosis, treatment sensitivity (radiotherapy, chemotherapy, and immunotherapy), and prognosis, depending on the signature of m6A-modified RNAs (mRNA, ncRNA) and status of m6A regulators (expression and mutation). This quantum leap from molecular mechanism to clinical application is of great significance for patient triage, identification of high-risk patients, and adjustment of treatment strategies.

Today, m6A is being revolutionized as a biomarker and potential therapeutic target in clinical practice. In many malignancies, m6A has shown good predictive efficacy in terms of diagnosis [109], treatment sensitivity (radiotherapy [110], chemotherapy [111], and immunotherapy [112]), and prognosis [113], depending on the signature of m6A-modified RNAs (mRNA, ncRNA) and status of m6A regulators (expression and mutation). The potential applications of m6A modification as a biomarker in CC are shown in Fig. 5. Notably, abnormal m6A modifications are equally strongly associated with tumor progression and prognosis in cancer patients. Therefore, targeting m6A modification regulators is also a promising and potential cancer treatment strategy.

### m6A as biomarkers

#### Diagnostic biomarkers

CC is the only malignancy with a clear cause, and it usually takes 10 to 20 years from the beginning of HPV infection to the development of CC. Therefore, the early diagnosis and treatment of cervical diseases are vital for eliminating CC. Currently, the most classic screening method of CC in the world is HPV tests combined with thin-prep cytology tests (TCT). With the advent of genomics, proteomics, and high-throughput sequencing [114] technologies, more and more new biomarkers are being discovered and used for diagnosing and typing diseases.

Using GEO and TCGA data of CC patients, Wang et al. comprehensively analyzed 33 m6A regulators and their indicator roles in diagnosing CC. They built a CC diagnosis model based on random forest (RF), support vector machine (SVM), and artificial neural network (ANN) models. This model emphasized the crucial diagnostic value of RBM15, NSUN2, HNRNPA2B1, METTL3, CBLL1, ELAVL1, RBMX, ABCF1, FXR1, YTHDF3 in CC, and can be applied in clinical practice to achieve remarkable accuracy (AUC = 0.999) [115].

#### Predictive biomarkers

The sensitivity of cancer patients to radiotherapy, chemotherapy, and immunotherapy determines the efficacy of treatment, which depends on the TME [116]. In recent years, convincing evidence has shown that m6A regulators and m6A modification levels on specific key transcripts can achieve remodeling of the TME, which are associated with local metabolic pathways [116], immune cell activation and infiltration [117], cell stemness [118] and immune escape [119] of cancer cells, resulting in resistance to therapy.

Du et al. found that YTHDF3 expression was upregulated in CC and positively correlated with the radio-resistance of cancer cells [21]. Wu et al. explored the influence of m6A on the reactivity of cisplatin drugs in CC and found that silencing YTHDF2 could enhance the sensitivity of Hela cells to cisplatin chemotherapy [20]. Zhou et al. found that overexpression of FTO was associated with resistance to chemoradiotherapy in CSCC, suggesting that patients with high expression of FTO were less likely to respond to CCRT despite being first-line treatment [22]. ICIs have ushered in a new dawn in CC, in which programmed cell death protein ligand 1 (PD-L1) expression plays a crucial role in immunotherapy reactivity. Excitingly, bioinformatics advances the process of quantitative analysis of tumor immune microenvironments. Analysis showed that m6A regulators were crucial mediators of PD-L1 expression and immune cell infiltration and could be used as a new biomarker to predict the efficacy of immunotherapy [120–122].

#### Prognostic biomarkers

As the biological role of chemical modifications in various diseases has been widely explored, non-mutational epigenetic reprogramming has become the hallmark of cancer in the 2020 s [59]. As the most widely modified type of RNA, m6A regulators have been considered to be one of the most promising prognostic biomarkers [123] because their expression and mutation status, as well as the characteristics of the modified RNA, and are strongly correlated with disease-free survival (DFS) and overall survival (OS).

In CC, METTL3 [31], METTL14 [124], and YTHDF1 [19] were overexpressed and associated with poor prognosis. While, ALKBH5 [47] was low expressed in CC, and predicted a poor prognosis. Studies showed that FTO was correlated with patients’ stage, and high FTO levels indicated the late FIGO stage of patients, which represented adverse outcomes for patients to some extent [72]. With the application of bioinformatics in medical research, comprehensive prediction models integrating multiple m6A regulators have been developed and validated. For example, Ji et al. developed a risk score system based on three m6A regulators, METTL16, YTHDF1, and ZC3H13, which showed good predictive performance in the prognosis of patients with CC [120]. Liu et al. and Zhang et al. screened out 10 and 6 m6A-related lncRNA to establish risk scores and verify the correlation between the model and disease prognosis, respectively [122, 125]. In addition, Ni et al. combined METTL3 and CD33^+^ myeloid-derived suppressor cells (MDSCs) in tissues to comprehensively predict the prognosis of CC patients [126]. Lu et al. combined m6A regulators with clinicopathological features, and the c-index showed good differentiation [115].

### m6A as therapeutic targets

As previously stated, many m6A regulators were overexpressed in CC and closely related to tumor cells’ stemness, immune escape, and resistance. Therefore, targeting m6A regulators has potential therapeutic significance in reducing tumor focus, and preventing metastasis. Over the years, through rational design, virtual screening, and structure-activity relationship studies by scientists, many specific and compelling inhibitors targeting the m6A mechanism have been developed and validated. For example, FTO, METTL3, IGF2BP2 small molecule inhibitors such as Rhein [127], STM2457 [128], CWI1-2 [129] have been preliminarily demonstrated in malignant tumors.

For advanced CC, targeted m6A inhibitors in combination with radiotherapy, chemotherapy, or immunotherapy can potentially improve outcomes in patients who have developed resistance to cisplatin and ICIs. More promising are nanocarrier-mediated drugs, which can target binding receptors while releasing drugs in a controlled manner. Compared with traditional drugs, nanomedicine can deliver drugs to target tissues or organs in a targeted manner to increase drug bioavailability, reduce drug toxicity, and increase drug solubility, thus enhancing the therapeutic effect [130–132]. You et al. engineered small extracellular vesicles (sEVs) with high CD47 expression to effectively deliver short interfering RNA against m6A reader YTHDF1 to treat gastric cancer via epigenetic and immune regulation [133]. Song et al. prepared a bimetallic metal-organic framework (MOF)-based biomimetic nanoplatforms (termed AFMMB), which could not only recover the STING pathway by inhibiting the DNA methylation but also inactivate endogenous iron-dependent m6A demethylase due to Fe^3+^ accumulation, thus increasing the overall m6A modification of RNA and inhibiting PD-L1 to achieve dual epigenetic therapy against leukemia [134]. However, there is still a lack of evidence for the treatment of m6A-targeted drugs in CC. Therefore, the application of current m6A-related small molecule inhibitors combined with nanotechnology in the clinical treatment of CC or the development of more m6A regulatory protein inhibitors or agonists with high specificity, selectivity, and safety will contribute to the progress of precision medicine based on RNA and methylation.

## Conclusions and perspectives

CC is the second most common malignant tumor among women, seriously threatening health and quality of life worldwide [135]. The World Health Organization (WHO) approved a global strategy of papillomavirus vaccination, screening, and precancerous and cancer treatment in May 2020 to support the elimination of CC as a public health problem [1]. Nevertheless, health inequalities based on ethnic, geographical, and economic differences will be one of the most significant challenges in achieving the goal of elimination [136]. More seriously, the COVID-19 pandemic that swept the world in 2019 has widened screening and treatment coverage inequalities, exacerbating barriers to eliminating CC [137].

The living environment of malignant tumor cells and their interaction determine the characteristics of tumor proliferation, differentiation, metastasis, and drug resistance. As the most extensive type of RNA epigenetic modification, m6A plays a vital role in the TME, which cannot be ignored. For example, m6A demethylase FTO, which is upregulated in primary and 5-fluorouracil (5-FU)-resistant colorectal cancer tissue, induced mRNA degradation by targeting SIVA1 in recognition of YTHDF2 [138]. In breast cancer, METTL3 induced high levels of m6A modification of LATS1 mRNA, and YTHDF2 reduced its stability by recognizing m6A sites in LATS1 mRNA, which was involved in glycolysis and tumor progression in breast cancer by inhibition of YAP/TAZ in Hippo pathway [139]. Similarly, studies of m6A in CC have mushroomed. Therefore, in this review, we introduce the abnormal expressions of m6A regulators, their mechanisms, effects on proliferation, metastasis, metabolic reprogramming, apoptosis in TME, and clinical applications.

For patients with recurrent, persistent, or metastatic CC, CCRT is limited in improving the prognosis. In the past few years, targeted therapy, namely bevacizumab, an anti-VEGF drug, in combination with platinum-based chemotherapy, has successfully extended the median survival of advanced CC to nearly 17 months [140]. The recent inclusion of immunotherapies such as ICIs in the first-line treatment of platinum therapy failure and persistent, recurrent, metastatic CC is a breakthrough. It should be emphasized that patients’ choice of immunotherapy approaches remains challenging. For example, the status of PD-L1 is critical to the efficacy of ICIs. However, according to the study, PD-L1 expression in CSCC varies greatly (from 19 to 88%) and is less expressed in cervical adenocarcinoma (14%) [141], which determines the individual differences and selectivity of pembrolizumab (anti-PD-1 monoclonal antibody) to improve survival. Sufficient studies have shown that m6A is related to immune cell infiltration, activation, and PD-L1 expression in TME of many cancers. Chen et al. found that the silencing of METTL3 in colorectal cancer cells could reduce the accumulation of MDSCs through the m6A-BHLHE41-CXCL axis to maintain the activation and proliferation of CD4^+^ and CD8^+^ T cells and eventually inhibited colorectal cancer [142]. In intrahepatic cholangiocarcinoma (ICC), ALKBH5, as an essential m6A demethylase, regulated the m6A modification in the 3’UTR region of PD-L1 mRNA, thereby inhibiting its degradation in a YTHDF2-dependent manner. The new regulatory mechanism of PD-L1 modification by ALKBH5 through mRNA epigenetic modification and the potential role of m6A in immunotherapy response were revealed [143]. Regrettably, there are no studies on m6A modification in the immune microenvironment of CC. However, the development of bioinformatics has explained that m6A is strongly correlated with immune cell infiltration and PD-L1 expression in CC, suggesting the potential role of m6A in immune escape and treatment [115, 120].

The detection of gene methylation levels, such as SOX1, PAX1, and JAM3, has been widely used in the early screening of cervical diseases and prognosis prediction [144, 145]. DNA methylation test kits have been developed and entered the clinical practice to perform shunt for low-grade squamous intraepithelial lesion (LSIL) and high-grade squamous intraepithelial lesion (HSIL) patients. Besides, cervical adenocarcinoma, one of the histological subtypes of CC, is less detectable by cytologic tests, and most cases show advanced disease at diagnosis. As a new biomarker, whether m6A can be used as a new screening method for CC is also worthy of in-depth exploration by gynecologists. In a word, we have obtained a deeper insight into CC based on m6A-related functions and molecular mechanisms, and will eventually improve the treatment effect and the life quality of patients with the applications of various advanced medical technologies.

### Supplementary information

Publication License
